# STX140, but Not Paclitaxel, Inhibits Mammary Tumour Initiation and Progression in C3(1)/SV40 T/t-Antigen Transgenic Mice

**DOI:** 10.1371/journal.pone.0080305

**Published:** 2013-12-06

**Authors:** Florence Meyer-Losic, Simon P. Newman, Joanna M. Day, Michael J. Reed, Philip G. Kasprzyk, Atul Purohit, Paul A. Foster

**Affiliations:** 1 IPSEN Innovation, IPSEN, Paris, France; 2 Oncology Drug Discovery and Women's Health Group, Imperial College London, London, United Kingdom; 3 Ipsen Biomeasure, IPSEN, Milford, Massachusetts, United States of America; 4 Centre for Endocrinology, Diabetes and Metabolism, School of Clinical and Experimental Medicine, University of Birmingham, Birmingham, United Kingdom; University of South Alabama, United States of America

## Abstract

Despite paclitxael's clinical success, treating hormone-refractory breast cancer remains challenging. Paclitaxel has a poor pharmacological profile, characterized by a low therapeutic index (TIX) caused by severe dose limiting toxicities, such as neutropenia and peripheral neuropathy. Consequently, new drugs are urgently required. STX140, a compound previously shown to have excellent efficacy against many tumors, is here compared to paclitaxel in three translational *in vivo* breast cancer models, a rat model of peripheral neuropathy, and through pharmacological testing. Three different *in vivo* mouse models of breast cancer were used; the metastatic 4T1 orthotopic model, the C3(1)/SV40 T-Ag model, and the MDA-MB-231 xenograft model. To determine TIX and pharmacological profile of STX140, a comprehensive dosing regime was performed in mice bearing MDA-MD-231 xenografts. Finally, peripheral neuropathy was examined using a rat plantar thermal hyperalgesia model. In the 4T1 metastatic model, STX140 and paclitaxel significantly inhibited primary tumor growth and lung metastases. All C3(1)/SV40 T-Ag mice in the control and paclitaxel treated groups developed palpable mammary cancer. STX140 blocked 47% of tumors developing and significantly inhibited growth of tumors that did develop. STX140 treatment caused a significant (P<0.001) survival advantage for animals in early and late intervention groups. Conversely, in C3(1)/SV40 T-Ag mice, paclitaxel failed to inhibit tumor growth and did not increase survival time. Furthermore, paclitaxel, but not STX140, induced significant peripheral neuropathy and neutropenia. These results show that STX140 has a greater anti-cancer efficacy, TIX, and reduced neurotoxicity compared to paclitaxel in C3(1)/SV40 T-Ag mice and therefore may be of significant benefit to patients with breast cancer.

## Introduction

Use of microtubule-targeting agents, such as the taxanes, to treat early- and late-stage breast cancer is now common. However, taxane efficacy is compromised by dose-limiting toxicities such as neutropenia and neurotoxicity. Hypersensitivity reactions are also triggered by the presence of non-ionic surfactant, Cremophor® EL, required in taxane formulations [1]. These factors result in poor physiological tolerance, and optimal anti-tumor doses can only be administered intermittently. Consequently, patients on taxane therapy require frequent treatment holidays which allows tumor regrowth and taxane resistance to develop. Although there has been some success in developing new taxane analogs, notably nab-paclitaxel and cabazitaxel, recent strategies aimed at lowering taxane associated toxicities have shown only limited success [2]. Therefore, there remains an unmet clinical requirement for compounds with both excellent efficacy against breast cancer and a reduced toxicity profile.

**Figure 1:**
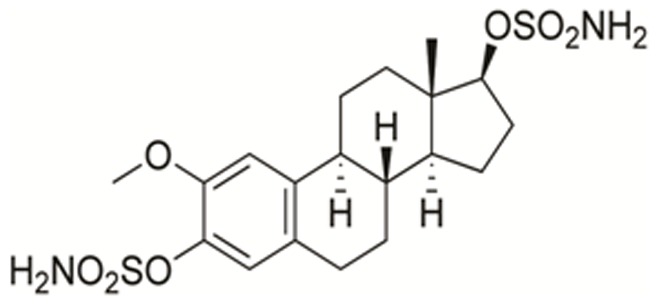
The chemical structure of STX140.

One such compound is STX140 (2-methoxyestradiol-*bis*-sulfamate; Figure 1) which shows oral efficacy against a range of *in vitro* cancer cell lines and *in vivo* xenograft tumor models [3]–[16]. Our previous work demonstrates that in breast cancer cells STX140, as measured by tubulin binding assays [4] and as observed in MCF-7 cells using immunohistochemistry [17], causes microtubule disruption and therefore tubulin destabilisation. This disruption results in elevated cell cycle arrest [4], mitochondrial uncoupling [11], release of cytochrome c [11], increased cyclin B1 expression [8], and phosphorylation of Bcl-2 [13], all leading to significant increases in cellular apoptosis. Therefore, STX140 and paclitaxel both target cell cycle mechanics by disrupting tubulin. Furthermore, STX140 also inhibits *in vitro* and *in vivo* tumor angiogenesis [6], [10], and has excellent oral bioavailability (at 87%) [7], [14], [15]. Furthermore, this compound is active *in vivo* against taxane-resistant breast cancer [13], and its efficacy *in vitro* is not impaired by known clinical mechanisms of resistance, such as the P-glycoprotein pump [13], BCRP [13], and βIII tubulin over-expression [17].

Here we compare STX140 efficacy with the taxane, paclitaxel, in C3(1)/SV40 T/t-antigen transgenic mice bearing hormone-independent breast cancer. This model recapitulates important histopathological and molecular alterations observed in human mammary cancer development over a highly predictable time-course [18]. Therefore, this approach has significant advantages over xenograft models, which have been much criticised over their failure to provide accurate predictions of drug efficacy in clinical trials [19], [20] Furthermore, C3(1)/SV40 T/t-antigen mice have been used extensively for the pre-clinical evaluation of anti-cancer agents [21]–[25]. To prepare for Phase I trials, we also investigate the toxicological and neuropathic profile of STX140 compared to paclitaxel in a mouse model of peripheral neuropathy. Finally, we determine STX140's therapeutic index (TIX), a key measurement to determine this compounds therapeutic window in clinical trials.

## Materials and Methods

### Ethics statement

Animals were housed according to local animal care protocols at Imperial College London, U.K. and at Ipsen, France, and complied with each countries animal welfare policies. Protocols for studies were approved by a UK Home Office Licence (PPL 70/6822). Animals lived on a 12∶12 hr light-dark cycle at 22°C, with access to food and water *ad libitum*. To limit suffering and for ethical reasons, in all studies animals were removed if total tumor mean diameter exceeded 15 mm, as recommended by Workman et al. [26].

### Compounds and materials

Details of the synthesis of STX140 have been published elsewhere [9]. Micronized STX140 was prepared in 0.5% methyl cellulose (MC; 400 cP). All other materials and medium were obtained from Sigma-Aldrich, U.K. unless otherwise stated.

### Cell culture

MDA-MB-231 cells and 4T1 mouse mammary cancer cells from the American Type Culture Collection (LGC Promochem, Teddington, U.K.) were grown in 10% foetal calf serum supplemented RPMI-1640 medium.

### Animals

Female athymic MF-1 mice aged 6–8 weeks were purchased from Harlan, UK. NCr-nude mice aged 6–8 weeks were purchased from Charles River, France. C3(1)/SV40 T/t-antigen heterozygous transgenic mice in the FVB/N background were bred from cryopreserved embryos obtained from the Jackson Laboratory (Maine, U.S.A.). Using tail snips mice were genotyped with primers for T-Ag:

5′-CAGAGCAGAATTGTGGAGTGG-3′, and

5′-ACAAACCACAACTAGAATGCAGTG-3′

Only female mice heterozygous for the C3(1)/SV40T-Ag transgene were used.

### Orthotopic 4T1 metastatic mouse model

STX140 and paclitaxel efficacy on metastatic tumor growth was assessed using the well characterised 4T1 cell mouse model [27]. 4T1 cells (2.5×10^6^) were inoculated into mammary fat pads of athymic MF-1 female mice. Once primary tumors had reached 100 mm^3^ animals were divided into three groups of ten: vehicle control (100 µl 0.5% MC p.o. five times weekly), STX140 (20 mg/kg p.o. five times weekly), and paclitaxel (15 mg/kg i.v. twice weekly). Using electronic callipers, tumor measurements were taken twice weekly and animal weights once weekly. Tumor volume (V) was calculated using the formula: (*length*×*width^2^*/2). Mice were culled 21 days after start of dosing and blood (via cardiac puncture), lungs and livers collected to determine circulating tumor cells (CTCs) and tissue metastasis.

### Quantification of 4T1 CTCs and metastases

#### Blood

Triplicate 30 µl aliquots of heparinized whole blood samples were mixed with 10% FBS in DMEM and plated in 6-well plates in the presence of 60 mM 6-thioguanine (6-TG). This selects for 4T1 cells as they are resistant to 6-TG-induced apoptosis [27]. After 72 hours at 37°C, plates were washed twice with HBSS, and cultured in 6-TG-supplemented 10% FBS in DMEM for 96 hours.

#### Lung and Liver

Freshly isolated livers were washed in HBSS, finely minced in 2 mg/ml filter-sterilized Collagenase I, and then placed at 37°C in 2 mg/ml Hyaluronidase for 20 min with agitation. Fresh lungs were washed in HBSS and finely minced in 5 IU elastase/1 mg/ml filter-sterilized Collagenase IV in HBSS. Minced samples were incubated at 4°C for 60 min with agitation. Lung and liver samples were then filtered through 70 µm nylon cell strainers (BD Falcon Cell Strainers; BD Biosciences, Oxford, U.K.). These filtered solutions were centrifuged at 1000 g for 5 min, the pellets washed with HBSS, re-suspended in 10% FBS in DMEM containing 60 µM TG, and aliquotted into 6-well plates (3 ml/well, 6 wells/sample). Plates were then placed in a 37°C incubator for 7 days.

After 7 days, plated 4T1 cells were incubated with methanol for 5 minutes and then stained with 0.04% Giemsa solution. 4T1 cell growth was quantified by scanning the stained plates using a UMAX Astra 4000U scanner (Umax UK Ltd., Milton Keynes, U.K.), and analyzing the mean intensity of the stain in each well using Kodak 1D software (Kodak Ltd., Hemel Hempstead, U.K.). Clonogenic metastases were calculated on a per-organ basis as an average mean intensity per well.

### C3(1)T-Ag Transgenic mouse studies and experimental scheduling

**Figure 2:**
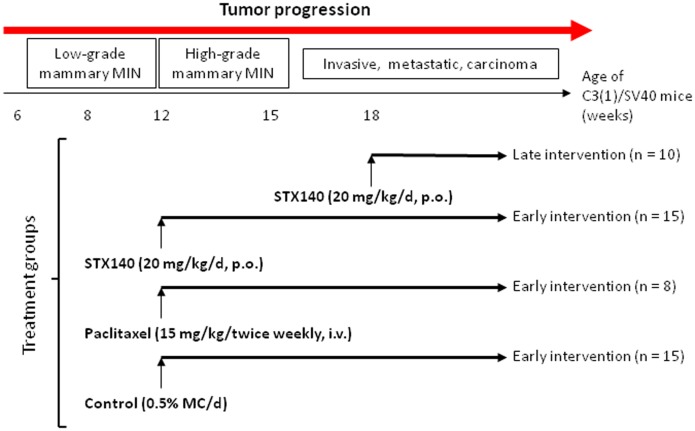
Experimental design for the use of STX140 and paclitaxel *in vivo* in C3(1)/SV40 Tag transgenic mice. Two different studies were performed. In the early intervention study, animals were treated at 12 weeks of age for 15 weeks; mice were euthanized if tumor length reached 1.5 cm in any direction. The late intervention experiment was initiated when the tumor burden was greater than 100 mm^3^ and dosing of STX140 was continuous until humane endpoints reached.

At 5 weeks of age, C3(1)T-Ag mice were randomly assigned into four separate groups: Control vehicle treated (0.5% MC p.o. daily, n = 15); Early intervention (dosing started at 12 weeks of age) STX140 (20 mg/kg/d, p.o., n = 15) and paclitaxel (15 mg/kg twice weekly i.v., n = 8); Late intervention (dosing started when tumor burden was greater than 100 mm^3^, approximately 20 weeks of age) STX140 (20 mg/kg/d, p.o. n = 10). For clarity, this treatment regiment is shown in Figure 2.

Mammary tumor measurements and body weights were taken weekly. Tumor size was measured using electronic callipers, and the tumor volume (V) calculated as previously mentioned. When animals presented with multiple tumors, volumes were added together to give total tumor burden. Mice were sacrificed if tumor burden reached greater than 2000 mm^3^ or if animals lost over 10% body weight.

### Peripheral Neuropathy

Determining mouse hind-paw thermal hyperalgesia is a well established method to measure drug induced peripheral neuropathy [28]. This method exposes the lateral plantar surface of the hind paw to a beam of radiant heat through a transparent Perspex surface using a plantar analgesia meter for paw stimulation [29]. Withdrawal latency to the heat stimulus is recorded for both left and right hind paws as the time taken from the onset of the thermal stimulus to withdrawal of the hind paw from the heat source. For each MF-1 female nude mouse, heat stimulation was repeated three times with an interval of 3 minutes between readings. Studies were performed double-blind. Animals were split into three groups of twelve: Vehicle (0.5% MC, p.o. daily), STX140 (20 mg/kg, p.o. daily), and paclitaxel (15 mg/kg, i.v. twice weekly) and withdrawal latencies measured twice weekly. At the end of the study, cardiac punctures were performed on all animals and circulating leukocyte counts ascertained via flow cytometry.

### Therapeutic Index (TIX) and PK analysis

MDA-MB-231 breast cancer cells were implanted (5×10^6^ cells/animal) s.c. into female NCr-nude mice on day 1. Once tumors reached a mean of 100 mm^3^, animals were randomly assigned to different groups to receive STX140 doses (10–80 mg/kg) and schedules (daily or intermittently, i.e. thrice weekly). STX140 was administered orally in 0.5% MC. Tumor volumes and body weights were recorded weekly. Blood samples were taken via tail vein bleeds on day 1 and day 28 of treatment for PK analysis. AUC was measured based on STX140 plasma levels. TIX was calculated based on first active and first toxic doses and the respective related exposures.

To determine compound neurotoxicity, female nude mice received either daily oral treatment with STX140 at the indicated doses or i.v. paclitaxel at 15 mg/kg twice weekly. At the end of the treatment period (day 43) or when the animals were found moribund, sciatic nerve, brain and lumbar region of the spinal cord were taken for anatomopathological analyses. Tissues were paraffin embedded, sectioned and H&E stained. Pathological assessment was performed by a professional anatomopathologist.

### Statistical Analysis

One-way ANOVA followed by a Bonferroni's multiple comparison test was performed to determine statistical significance where applicable. Where only two groups are compared a Student's *t* test was applied. All values are represented as the mean ± standard error of the mean (SEM). For all survival data the difference in the survival distributions of two samples was determined using the Mantel-Cox test.

## Results

### STX140 and paclitaxel inhibit 4T1 xenograft tumor growth and metastasis

**Figure 3:**
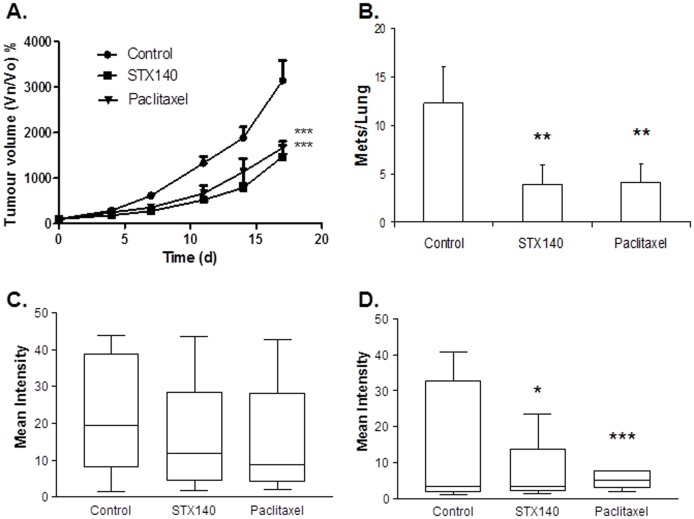
The effect of STX140 (20 mg/kg/daily p.o.) and paclitaxel (15 mg/kg/twice weekly i.v.) on 4T1 orthotopic tumor growth and metastasis. A) STX140 and paclitaxel inhibit primary mammary carcinoma growth. B) Counts of the number of metastatic lesions on the lungs of animals treated with STX140 and paclitaxel. C) 4T1 colonies cultured from the lungs of these mice. D) 4T1 colonies cultured from the blood of mice bearing 4T1 mammary tumors. Data represent mean ± s.e.m. * P<0.05, ** P<0.01, *** P<0.001 compared to controls. n = 8/group.

Control tumors developed rapidly and were 1572±220 mm^3^ at day 17 post-inoculation. STX140 and paclitaxel significantly (P<0.05) inhibited primary orthotopic mammary tumor growth (Fig. 3A) with no effect on mouse weights (not shown). At day 17, blood samples, liver and lung tissue were collected to assess plasma CTC levels and metastatic lesions. Upon dissection lungs were observed to contain numerous cancerous lesions. There were significantly more metastatic lesions (p<0.01) in vehicle treated animals compared to STX140 and paclitaxel treatment (Fig. 3B). Digestion and culturing cells from lung tissue, with selection for 6-TG-resistant 4T1 colonies, also demonstrated a reduction in the number of lung metastases as a result of STX140 and paclitaxel treatment compared to control (Fig. 3C). This reduction was also observed in CTCs. Figure 3D shows the number of colonies that formed from 30 µl of blood after 10 days culture in 6-TG.

### STX140 and paclitaxel in the C3(1)/T-Ag transgenic mouse

Experimental schematics for these studies are shown in Figure 2. The early intervention study assessed STX140 efficacy against paclitaxel in this model. Unfortunately, not enough heterozygous C3(1)/SV40 T/t-antigen mice were bred to compare STX140 against paclitaxel in the late intervention arm.

### Early Intervention

**Figure 4:**
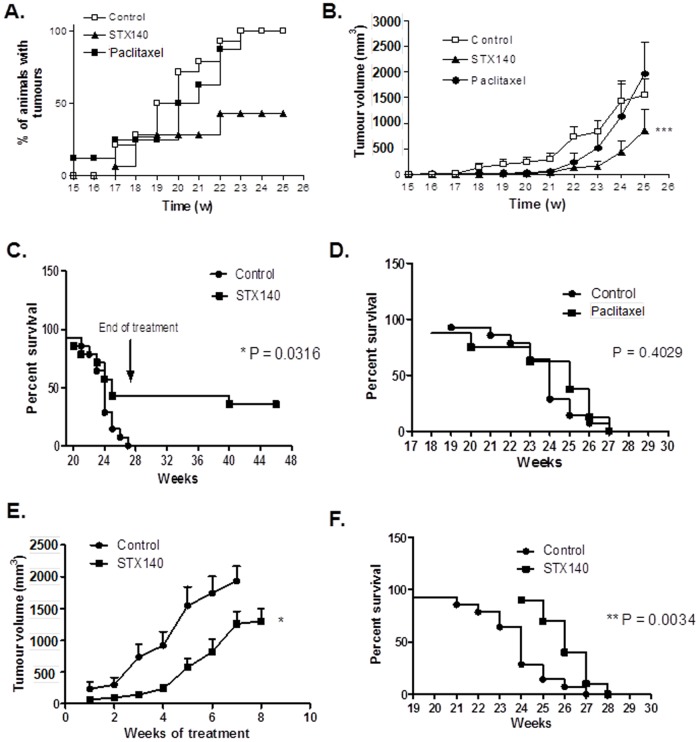
The effect of STX140 (20 mg/kg/daily p.o.) and paclitaxel (15 mg/kg/twice weekly i.v.) on tumor development in C3(1)/SV40 T-Ag mice. A) Palpable tumors developed in all control and paclitaxel treated animals, but not in the STX140 treated group. B) When administered at 12 weeks of age (early intervention), STX140 inhibited development of the overall tumor burden throughout the study. C) and D) STX140, not paclitaxel, treatment (early intervention) gave a significant survival advantage to C3(1)/SV40 T-Ag mice. E) During the late intervention stage of the study, treatment with STX140 commenced when tumor burden was greater than 100 mm^3^. STX140 reduced tumor burden in these animals and F) conferred a significant survival advantage. Data represent mean ± s.e.m. * P<0.05, ** P<0.01, *** P<0.001 compared to controls. n = 10–15/group.

Palpable mammary tumors were evident after 14 weeks and all animals in the control group presented with malignancies by the 23^rd^ week of age. Figure 4A shows that the onset of palpable tumors was significantly attenuated by STX140 (P<0.001), but not paclitaxel. Similar to the control group, paclitaxel-treated animals all developed mammary carcinoma by the 23^rd^ week. In contrast, in 47% of mice treated at week 12 onwards with STX140, no palpable tumors were observed throughout the study.

In vehicle-treated animals, mammary cancer growth was first observed at 17 weeks of age. Cumulative tumor burden reached 1556.6±289.8 mm^3^ by week 25. Tumor growth rate, shown in Figure 4B, was initially significantly inhibited by both STX140 and paclitaxel (P<0.001). However, by week 23, paclitaxel treatment did not significantly reduce total tumor burden (at 519.7±280.6 mm^3^) compared to controls (at 829.9±222.6 mm^3^). In mice that did develop malignancy, mammary tumor growth was significantly (P<0.001) inhibited with STX140 treatment throughout the treatment period (weeks 12–25), reaching a cumulative tumor burden volume of 852.9±425 mm^3^ at week 25.

To manage and reduce animal suffering, once animals had either lost 10% of their body weight or tumor burden exceeded 2000 mm^3^, they were humanely culled and survival data calculated (Figures 4C and 4D). All vehicle treated mice were terminated by the 27^th^ week of age. This was due exclusively to excessive tumor burden. Paclitaxel treatment had no effect on animal survival, with all animals removed from the study by week 27. In contrast, STX140 dosing (p.o. daily from weeks 12–27) resulted in significantly prolonged survival rates (P = 0.0316) with 43% of animals having no observable tumors throughout the study.

### Late intervention

Mice bearing tumors at 100–300 mm^3^ were treated with 20 mg/kg/d STX140 for 8 weeks (unless tumor burden reached over 2000 mm^3^ at which point animals were euthanized). Results are shown in Figure 4E. At the start of the experiment there was a non-significant difference in tumor size between the vehicle and STX140 treated animals (236.7±108.4 mm^3^ compared to 107.8±40.3 mm^3^). Three weeks dosing with STX140 resulted in a significant 80.4% reduction in tumor growth compared to controls and this reduction was 35.0% (P<0.05) after 7 weeks. Animals receiving STX140 demonstrated a small but significant (P<0.0034) survival advantage of 1–2 weeks compared to control animals (Figure 4F).

### TIX, PK profile, and neurotoxicity of STX140

**Figure 5:**
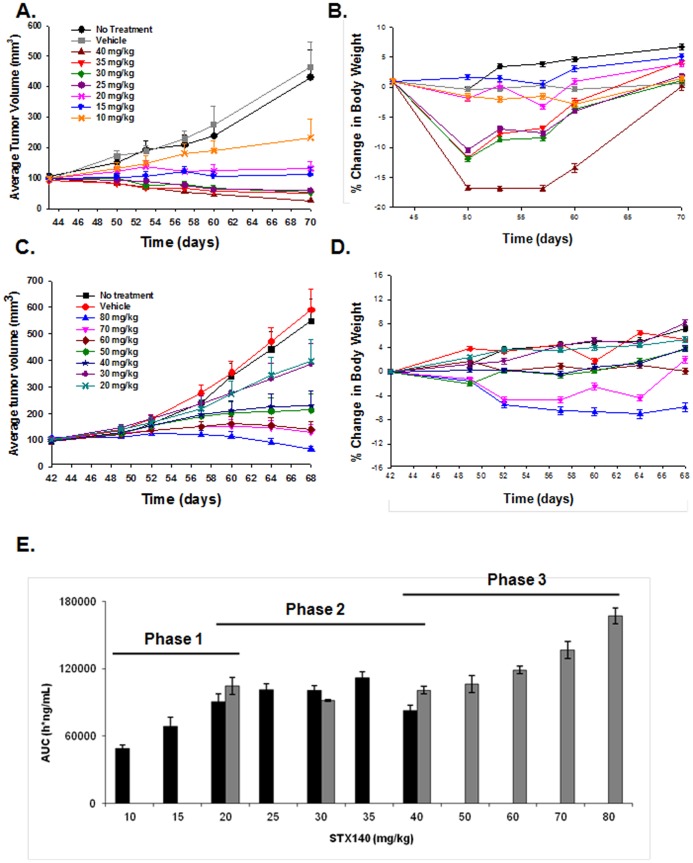
The spectrum of STX140 anti-cancer efficacy in mice bearing MDA-MB-231 breast cancer xenografts. Two dosing regimens were employed, daily (panels A and B), and thrice weekly (panels C and D). A) Daily dosing of STX140 was significantly efficacious at 10 mg/kg and caused animal mortality at 30 mg/kg. B) Animals treated with 40, 35, and 30 mg/kg initially showed signs of significant weight loss, although this response was not seen in all animals hence the apparent recovery throughout the study. C) Thrice weekly dosing of STX140 was significantly efficacious at 40 mg/kg, with mortality only observed at 80 mg/kg (1 out of 21 animals). D) Animals treated with 70 and 80 mg/kg STX140 showed some weight loss, however only 1 reached the humane endpoint of a greater than 10% reduction in body weight. E) Plasma concentration, as measured by AUC (ng/ml) of STX140 24 h after single dose. A three phase response is seen; firstly an initial dose-proportional increase (10–20 mg/kg), a secondary plateau (20–40 mg/kg), followed by a tertiary trend toward a dose-proportional increase (40–80 mg/kg). Data represents mean ± s.e.m. * P<0.05 compared to controls. n = 21/group.

In mice bearing MDA-MB-231 xenografts, four of the administered doses of STX140 (40, 35, 30, and 25 mg/kg/d) induced tumor regression (Fig. 5A). All daily dosing regimens (10–40 mg/kg) showed some efficacy (with an ED_50_ of 10 mg/kg/d), with 30 mg/kg/d being the lowest dose causing mortality (1 death in 21 mice), although 25 mg/kg/d did result in initial substantial weight loss (Fig. 5B). As the highest dose of STX140 (40 mg/kg/d) did not result in a 50% mortality rate, LD_50_ could not be ascertained. However, as 52.3% of mice treated with 30 mg/kg/d STX140 lost over 10% body weight, we have used this parameter as our LD_50_ marker.

On the intermittent schedule one dose induced MDA-MB-231 tumor regression (80 mg/kg thrice weekly, Fig. 5C, with an ED_50_ of 20 mg/kg thrice weekly), and this dosing regimen was better tolerated with less toxicity in terms of body weight loss and animal mortality (Fig. 5D). Using similar reasoning as mentioned above, we calculated the LD_50_ as 80 mg/kg thrice weekly. Consequently, TIX was calculated (*LD*50÷*ED*50) as:
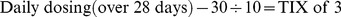


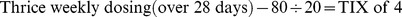
In terms of PK, no plasma accumulation of STX140 was observed, even after 28 days of continuous daily oral treatment (Fig. S1). No clear dose-proportionality could be observed (Fig. 5E), rather a 3 phase PK behavior with a first phase of dose-proportional increase between 10 and 20 mg/kg, then a second phase with a plateau (25 to 40 mg/kg) and finally a third phase with a trend to dose-dependent increase (40–80 mg/kg).

In neurotoxicity studies, paclitaxel demonstrated neurotoxic effect in both experiments, showing axonal degeneration in the sciatic nerves, minimal to mild degeneration within the white matter of the dorsal funiculus of the lumbar spinal cord (Table 1). In contrast, anatomopathological analyses of sciatic nerves, brain and lumbar region of the spinal cord of nude mice treated with STX140 revealed that, even at toxic doses, no lesions were observed, suggesting limited neurotoxic effect.

**Table 1:** Anatomopathological analysis of brain, spinal cord and sciatic nerve from nude mice treated with STX140 or paclitaxel.

Agent	mg/kg	Route	Schedule	Mortality	Neurotoxic
none	-	-	-	No	No
paclitaxel	30	iv	qod ×5	No	Yes
STX140	35	po	qd ×10	Yes	No
STX140	30	po	qd ×7	Yes	No
STX140	25	po	qd ×11	No	No
STX140	20	po	qd ×24	No	No
STX140	10	po	qd ×28	No	No

**Figure 6:**
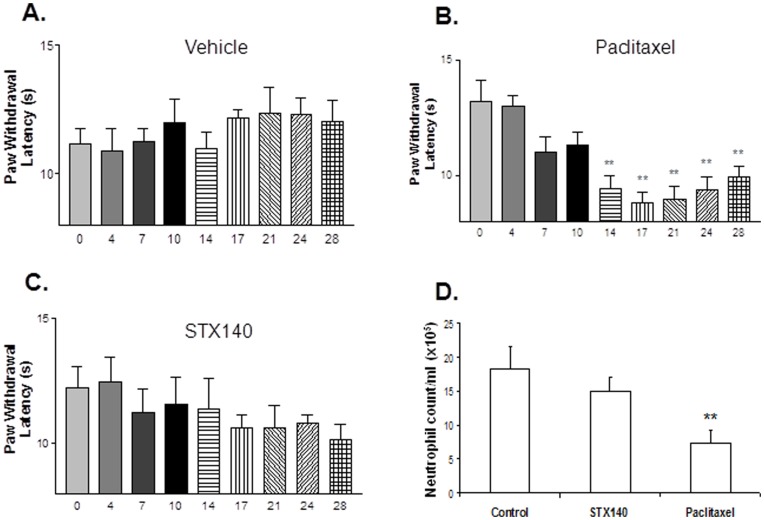
The effects of STX140 and paclitaxel on thermal paw withdrawal latency in MF-1 female nude mice. A) Vehicle treated control animals paw withdrawal did not alter throughout experiment. B) Twice weekly dosing of paclitaxel, by day 14, caused significant thermal hyperalgesia. C) Daily dosing of STX140 did not significantly affect paw withdrawal latencies. D) Plasma neutropenia was evident in animals treated with paclitaxel, but not with STX140. Data represents mean ± s.e.m. ** P<0.01 compared to day 0 from the same group. n = 10/group.

We further investigated STX140 neurotoxicity profile compared to paclitaxel using a thermal hyperalgesia method of peripheral neuropathy. Average paw withdrawal latencies in untreated animals was between 11–13 s (Fig. 6A). Daily treatment of vehicle or STX140 (Fig. 6A and 6C) did not affect withdrawal latency relative to day 0. However, animals treated twice weekly with 15 mg/kg (i.v.) of paclitaxel demonstrated reduced paw withdrawal latency (Fig. 6B). This significant neuropathy, at a withdrawal latency of 9.4±1.8 s, developed after 14 days of treatment (four paclitaxel doses). Furthermore, significant neutropenia was observed in blood samples obtained from paclitaxel treated animals, but not in STX140 treated mice (Fig. 6D).

## Discussion

Paclitaxel, despite possessing numerous toxicological challenges and inducing drug-resistance, remains the drug of choice for many oncologists. The early promise of overtly targeted therapies has yet to be realised and where efficacy is seen this is usually in combination with a well established cytotoxic regime. With new sequencing studies revealing the degree of heterogeneity and evolution occurring within individual tumours there is a new emphasise for multi-targeted agents which may act with or replace existing cytotoxic drugs [30]. Consequently, the identification of new compounds effective against both early- and late-stage breast cancer and have favourable toxicity profiles remains paramount to advancing treatment. Therefore, this work aimed to: 1) compare STX140 with paclitaxel on the growth, metastasis and survival outcomes of two stages of mammary cancer development in two different pre-clinically relevant mammary cancer mouse models and: 2) determine whether STX140 possesses a favourable TIX with an acceptable toxicity profile in regards to effects on axonal degeneration and circulating leukocytes.

Our initial studies focused on the highly aggressive, metastatic 4T1 breast cancer model. STX140 showed similar efficacy to paclitaxel, significantly reducing primary tumor growth and lung and liver metastatic lesions. Furthermore, both compounds reduced CTC numbers and metastatic tumor cells obtained from lungs. This is the first time STX140 has been shown to be effective at reducing metastasis, and therefore warranted moving this compound forward into more clinically relevant breast cancer models prior to Phase I studies.

Therefore, we next investigated STX140 efficacy in the C3(1)/SV40 T-Ag mouse model of breast cancer. In the early intervention arm, at 12 weeks of age when high-grade MIN (similar to human ductal carcinoma *in situ*) had developed, mice were treated with either STX140 or paclitaxel. In the STX140-treated group, 43% of the animals did not develop tumors, and it was only STX140 that significantly inhibited mammary cancer growth. Unexpectedly, paclitaxel only had anti-proliferative effects on early tumor growth, and no effect on blocking tumors becoming physically palpable.

In the late intervention study only STX140 was investigated. STX140 administered to animals with larger tumor burdens (100–300 mm^3^) significantly reduced burden leading to an increased survival outcome. These results are encouraging considering the aggressive nature of these cancer models and indicate that STX140 may provide clinical benefit to patients suffering from both early- and late-stage breast cancer.

Previous research in C3(1)/SV40 T-Ag mice showed 150 mg/kg/d 2-ME, of which STX140 is a derivative, also significantly inhibited breast tumor growth [25]. However, in the early intervention regimen, and in contrast to STX140, all 2-ME treated animals developed mammary cancer. 2-ME is rapidly inactivated in the gut by 17β-hydroxysteroid dehydrogenase type-2 [31], this most likely accounting for its poor PK profile. Consequently, high doses of 2-ME are required to maintain efficacious plasma concentrations. In contrast, STX140 avoids first-pass metabolism and is readily detected in plasma after a single 20 mg/kg oral dose [7], and this superior PK profile may account for STX140 proving more effective in C3(1)/SV40 T-Ag mice compared to 2-ME.

It is unclear why paclitaxel failed to completely inhibit mammary cancer growth in the early intervention C3(1)/SV40 T-Ag mouse study. To our knowledge, there are no reports of paclitaxel use in C3(1)/SV40 T-Ag mice. As paclitaxel was discovered and clinically developed prior to the availability of transgenic mouse models, only a handful of studies have examined its effects in these model systems. When administered to TgMISIIR-TAg-DR26 transgenic mice that develop spontaneous ovarian cancer, paclitaxel, in combination with cisplatin, significantly reduced tumor burden [32]. Furthermore, paclitaxel alone inhibited the growth of retinoblastoma in LH beta-Tag–positive mice [33]. Interestingly, in our study, paclitaxel did inhibit growth from week 19 to 22 in the early intervention arm of our study, with this inhibition being lost from week 22 onwards. Due to known dose limiting toxicities, as confirmed by our thermal hyperalgesia and neutropenia studies and shown elsewhere [34], [35], paclitaxel was only administered intravenously twice weekly at 15 mg/kg. This scheduling limits paclitaxel's plasma availability, and therefore tumor exposure time. Consequently, loss of efficacy may be the result of taxane-resistance or the limited exposure time may have compromised paclitaxel efficacy against the aggressive disease seen in these animals. In contrast, tumor growth continued to be suppressed by week 23 in STX140 treated animals, although after this time proliferation did increase.

Previous studies have demonstrated the excellent efficacy of STX140 against MDA-MB-231 xenografts [12], [14]. However, the TIX – vital to inform future clinical studies – of STX140 was unknown. To determine this we performed a large ranging study comprising of two dosing schedules – daily and thrice weekly – in mice bearing MDA-MB-231 breast cancer xenografts. These studies allowed for the determination of a TIX of 3 (for daily dosing), and 4 (for weekly dosing). Previous research has identified STX140 to have a maximum tolerated dose of 150 mg/kg (p.o.) [13], in contrast to paclitaxel at 80 mg/kg [36].

In conclusion, this new data on STX140 activity demonstrated that it confers significant tumor reduction and survival advantage to C3(1)/SV40 T-Ag mice with both early- and late-stage carcinomas. Orthotopic mammary cancer growth was also blocked by STX140 and paclitaxel, with both drugs inhibiting lung 4T1 cell metastasis. In thermal hyperalgesia studies, paclitaxel, but not STX140, increased animal paw withdrawal latencies indicative of peripheral neuropathy. Furthermore, STX140 possessed a TIX of 3–4, suggesting a compound with favourably clinical application. As a prototype STX140 oral tablet formulation has already been developed and tested in vivo [14], this promising compound is now ready to be moved into Phase I clinical trials for patients with hormone-independent cancers.

## Supporting Information

Figure S1 **STX140 (given orally at 10, 20, 40, 80 mg/kg/d) does not accumulate in mouse plasma after repeated administration, as measured on day 1 and day 28 post STX140 administration.**
(TIF)
