# Supplementary figures and images for: STX140, but Not Paclitaxel, Inhibits Mammary Tumour Initiation and Progression in C3(1)/SV40 T/t-Antigen Transgenic Mice
Source: PLoS One. 2013 Dec 6;8(12):e80305. doi: 10.1371/journal.pone.0080305 (PMC3855596; doi:10.1371/journal.pone.0080305)

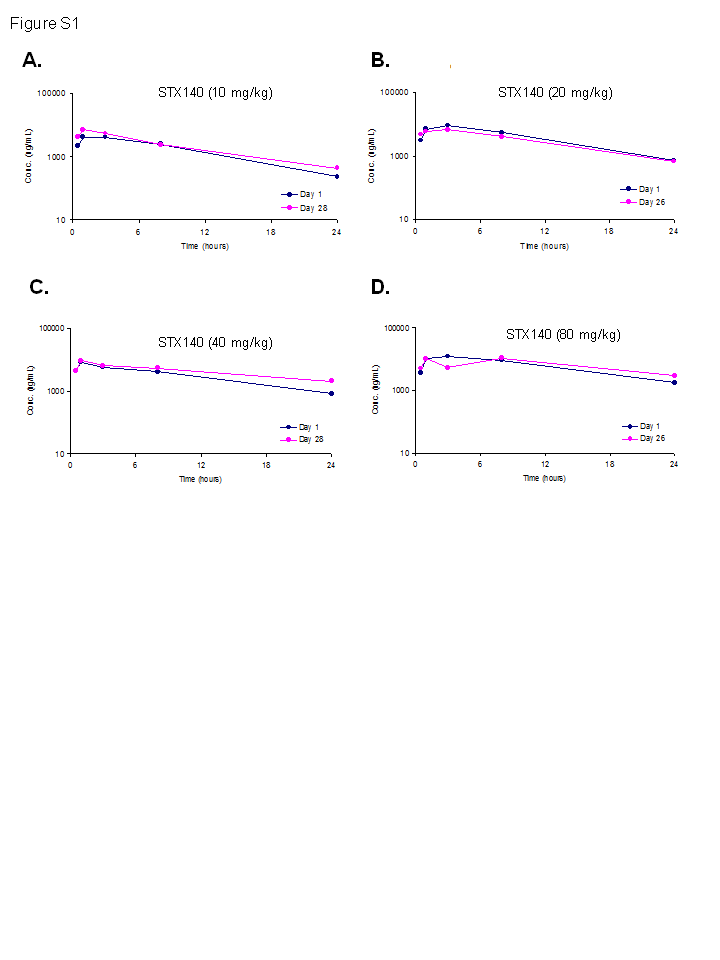

Supplement: Figure S1 — STX140 (given orally at 10, 20, 40, 80 mg/kg/d) does not accumulate in mouse plasma after repeated administration, as measured on day 1 and day 28 post STX140 administration. (TIF) [file pone.0080305.s001.tif]
